# Shaping axial identity during human pluripotent stem cell differentiation to neural crest cells

**DOI:** 10.1042/BST20211152

**Published:** 2021-01-11

**Authors:** Fay Cooper, Anestis Tsakiridis

**Affiliations:** 1 Centre for Stem Cell Biology, School of Biosciences, The University of Sheffield, Western Bank, Sheffield S10 2TN, U.K.; 2 Neuroscience Institute, The University of Sheffield, Western Bank, Sheffield S10 2TN, U.K.

**Keywords:** anterior–posterior patterning, cell differentiation, homeobox genes, human pluripotent stem cells, neural crest cells

## Abstract

The neural crest (NC) is a multipotent cell population which can give rise to a vast array of derivatives including neurons and glia of the peripheral nervous system, cartilage, cardiac smooth muscle, melanocytes and sympathoadrenal cells. An attractive strategy to model human NC development and associated birth defects as well as produce clinically relevant cell populations for regenerative medicine applications involves the *in vitro* generation of NC from human pluripotent stem cells (hPSCs). However, *in vivo*, the potential of NC cells to generate distinct cell types is determined by their position along the anteroposterior (A–P) axis and, therefore the axial identity of hPSC-derived NC cells is an important aspect to consider. Recent advances in understanding the developmental origins of NC and the signalling pathways involved in its specification have aided the *in vitro* generation of human NC cells which are representative of various A–P positions. Here, we explore recent advances in methodologies of *in vitro* NC specification and axis patterning using hPSCs.

## Introduction

Human pluripotent stem cells (hPSCs) have become a powerful resource to study embryonic development, circumventing both the technical and ethical obstacles associated with the use of human embryos. By harnessing the correct developmental signalling pathways during hPSC differentiation, it has become increasingly feasible to generate a vast array of human progenitor cell types with distinct identities and differentiation potential. Combined with the use of patient-derived induced pluripotent stem cells (iPSCs) or CRISPR/Cas9 genome editing, it is possible to use these hPSC derived progenitors in disease modelling or for therapeutic applications such as cell replacement/regenerative medicine therapies. The position of the various cell types that make up the emerging nervous system along the embryonic anteroposterior (A–P) axis is a critical determinant of their functionality, developmental potential and disease vulnerability and an increasing body of evidence has pointed out that this is also the case for their hPSC-derived counterparts. Thus, shaping the desired A–P axial identity *in vitro* is a crucial aspect in the design of hPSC neural differentiation protocols. The A–P regionalisation of hPSC-derived central nervous system (CNS)-associated cell types has already been discussed in detail in some excellent recent reviews elsewhere [1,2]. Here, we focus on recent findings on the key cellular and signalling parameters directing axial identity acquisition during the transition of hPSCs toward NC cells, the precursors of the peripheral nervous system.

## Formation and regionalisation of the nervous system *in vivo*

During mammalian embryonic development, the earliest cell lineage progenitors arise in the pluripotent post-implantation epiblast with the onset of gastrulation, which is marked by the emergence of a posterior structure known as the primitive streak (PS) under the influence of BMP, Wnt and Nodal signalling, followed by extensive morphogenetic movements leading to the continuous ingression of epiblast cells through the extending PS [3,4]. In turn, this leads to the rearrangement of the single epithelium epiblast into the three germ layers, which act as the building blocks of all embryonic tissues: ectoderm, mesoderm and endoderm. The central nervous system is derived from the ectoderm (neural plate) under the influence of an ‘organiser’ tissue possessing neural inductive properties, manifested though BMP, Wnt and Nodal signalling inhibition [5,6]. In amniote embryos, the principal neural-inductive organisers include the node (Hensen's node in the chick embryo), a region at the anterior tip of the PS, and axial mesoderm cells (its differentiated descendants); these have been shown in grafting experiments to induce a secondary body axis, including a nervous system, in pluripotent epiblast cells [7–11]. It is widely thought, based on early experiments in amphibians [12,13], that neural induction by an organiser, via BMP/Wnt/Nodal inhibition, leads by default to the generation of neural cells with anterior features (forebrain) (‘activation’). These anterior neural cells then become progressively ‘posteriorised’ under the influence of signals such as retinoic acid (RA) and WNT/FGF agonists to produce more posterior structures such as the hindbrain and the spinal cord (‘transformation’).

**Figure 1.**
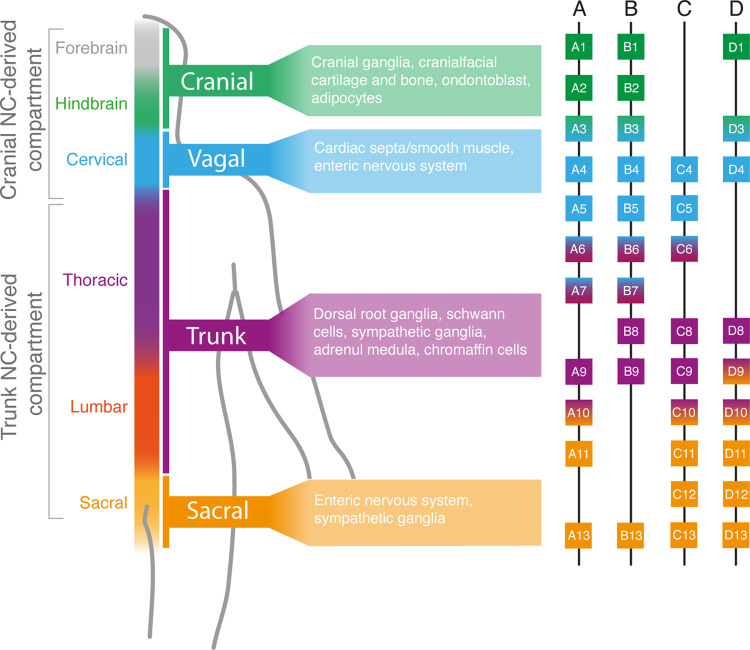
HOX gene expression in neural crest along the anterior–posterior axis. The A–P axis is defined by the combination of overlapping HOX(A–D) expression. Neural crest is divided into four main categories along the A–P axis based on *HOX* gene expression: cranial, vagal, trunk and sacral. Cranial neural crest (green) is divided into the anterior most HOX negative neural crest and the posterior cranial HOX positive neural crest, expressing HOX PG (1–3). Vagal neural crest (blue) express HOX PG (3–7) genes and are located parallel to the cervical region of the spinal cord. Trunk neural crest (purple) express HOX(6–9) genes are derived adjacent to the thoracic/lumbar region of the spinal cord. Sacral neural crest (orange), derived from the most posterior part of the A–P axis, express HOX (10–13 genes). The potential of NC cells to generate distinct cell types is determined by their position along the anteroposterior (A–P) axis and the major derivatives of each subpopulation is shown in the corresponding section.

Fate-mapping/clonal analysis experiments in the mouse embryo have revealed that the ectodermal precursors of brain components and spinal cord are found in distinct, defined locations in the late-gastrula mouse epiblast. These reflect the A–P regionalisation of their descendants i.e. the forebrain is derived exclusively from cells in the anterior epiblast whereas the spinal cord originates from a more distal site around the node [14–16]. By the end of gastrulation, all head neural structures in mammalian embryos have been laid whereas the production of the spinal cord continues throughout embryonic axis elongation via the addition of tissue in a head-to-tail direction. This process of post-gastrulation spinal cord construction appears to be distinct from the generation of the anterior (brain) parts of the central nervous system, consistent with a mode of growth driven by a transient pool of axial progenitors exhibiting stem cell-like properties [17–19]. These are bipotent and are marked by the ability to generate both spinal cord and presomitic mesoderm cells and are thus referred as neuromesodermal progenitors (NMPs). NMPs are located in a ‘posterior growth zone’ around the PS/node, including the node-streak border (NSB) and the caudal lateral epiblast (CLE) during early somite stages, and later in the PS-derived tailbud in a region known as the chordoneural hinge (CNH) (reviewed in [20–22]). Typically, NMPs are characterised by the co-expression of the nascent mesoderm marker Brachyury (Bra/T or TBXT in humans) and the neural marker SOX2, together with other posterior markers such as CDX2 and NKX1–2. NMP specification, maintenance and differentiation are orchestrated primarily via the interplay between the Wnt, FGF and retinoic acid (RA) signalling pathways [20–22]. The final A–P coordinates of the cellular parts of the fully formed nervous system are defined and determined by the combinatorial expression of Hox genes, which are arranged as paralogous groups (PG) (1–13) in four chromosomal clusters (A–D) [23] (Figure 1).

## The development of NC and its derivatives *in vivo*

The origin of NC is tightly linked with that of the central nervous system, specified at the neural plate border between the neural plate (future CNS) and the non-neural ectoderm (future epidermis). Initially, NC is induced by a combination of signals, primarily linked to the BMP, FGF and Wnt pathways [24,25] and these have been reviewed in depth elsewhere [26–31]. Combined signalling from these pathways induces the expression of key NC specifier transcription factors such as MSX1/2, PAX3/7 and ZIC genes which overlap at the future neural plate border [32–34]. These transcription factors, induce, in turn, definitive NC markers including SOX9/10, SNAI2 and FOXD3 forming a complex gene regulatory network which specifies and maintains NC cells [30,35,36].

The NC populates the neural tube along the entire A–P axis and can be subdivided into four main subregions along this axis: cranial, vagal, trunk and sacral. Their position along this axis determines their gene expression, migratory paths and subsequently their differentiation potential and contribution to the embryo. The cranial NC gives rise to the mesectoderm (e.g. cartilage and bone) in the head, melanocytes, sensory neurons and glia [37,38]. They are further divided into an anterior Hox negative and a more posterior Hox PG (1–3)-positive domain [39] (Figure 1). Parallel to the first seven somites of the neural tube, vagal NC express Hox PG (3–7) members and contribute to the enteric nervous system (ENS) and various heart structures [40–45]. Located between somites 8–24 (in mouse), the trunk NC contributes to the sympathetic nervous system, neuroendocrine cells, and melanocytes [46]. Trunk NC cells are characterised by the expression of more posterior Hox genes (Hox PG (6–9)) [43,47–49] (Figure 1). The region from somite 24 (in mouse) and below coincides with lumbosacral Hox PG 10–13 expression (Figure 1) and the presence of the sacral NC [50,51]. In amniotes, the sacral NC contributes to the most distal part of the enteric nervous system, unlike in zebrafish where the ENS is derived only from the vagal NC [52–56]. In addition to ENS, sacral NC contributes to the pelvic ganglia and therefore provides autonomic innovation of the lower urogenital tract [57].

Fate mapping and lineage tracing experiments have indicated a close link between NMPs and posterior NC precursors [58–62]. For example, homotopic grafting experiments of SOX2+/T+ cells taken from the E8.5 mouse CLE were found to result in NC contribution [59]. Similarly, lineage tracing of cells expressing NMP markers such as *Nkx1–2*, *Tbx6/Sox2*, *Bra/T* and *FoxB1* revealed that these contribute to neural, mesodermal and NC lineages [58,61,63–68]. More recent work, using single-cell transcriptomics and epigenomic profiling in zebrafish, has further demonstrated that posterior NC is derived from an NMP-derived pre-neural intermediate [69]. Taken together these data strongly suggest that, at least a fraction of trunk and sacral NC are directly derived from axial progenitors, which are competent to generate presomitic mesoderm.

## Development *in vitro*: generating NC cells of distinct axial identities from hPSCs

### Cranial NC

Early *in vitro* differentiation experiments focused on the induction of NC cells from a hPSC-derived neuroectodermal progenitor intermediate [70–72]. These initial protocols relied on recapitulating the signalling events which accompany neural induction *in vivo,* by blocking BMP and TGFB/Activin/Nodal signalling using corresponding small molecule inhibitors (termed dual SMAD inhibition) [70,73]. However, these protocols typically yielded a variable and low percentage of NC progenitors and as NC specification *in vivo* primarily occurs under the influence of intermediate BMP and Wnt signalling, subsequent studies found that a short pulse of dual SMAD-inhibition followed by the addition of BMP and Wnt signalling yielded a higher proportion of NC progenitors [74,75]. Further refinement and characterisation of these strategies revealed that hPSC-derived NC appears to emerge from a pre-neural border intermediate which relies on intermediate BMP and Wnt activity [76–78]. PAX6 expression (indicating neural progenitor specification) in these protocols occurred at the expense of the NC specifier SOX10 in line with *in vivo* findings indicating these cells may diverge at an earlier timepoint (reviewed further in [31]). Varying endogenous BMP signalling levels within the cells and/or BMP ligands in the media were found to affect overall BMP activity and lead to large variations in the efficiency in NC specification thus complicating the view of BMP requirement *in vitro*[79]. Furthermore, high BMP signalling can lead to specification of the cranial placode or non-neural ectoderm whereas low BMP can result in neuroectoderm specification [78]. As a result, more recent improved protocols which modulate BMP levels through top-down inhibition demonstrated that a precise level of intermediate BMP activity is required to specify NC efficiently and reproducibly from hPSCs *in vitro* [79,80]. A common feature of these approaches is that the NC cells they give rise to exhibit minimal *HOX* gene expression indicating a cranial axial identity. This is also supported by the demonstration that they can be further directed to differentiate toward peripheral neurons including (nociceptors, mechanoreceptors, and proprioceptors) and glia, melanocytes and mesenchymal lineages (smooth muscle, osteogenic and chondrogenic cells) [39,81].

### Vagal NC

Early efforts in the posterior patterning of human NC cells *in vitro* have been heavily influenced by Nieuwkoop's ‘activation-transformation’ model focusing on the production of an anterior neuroectodermal precursor followed by addition of NC promoting signals in combination with posteriorising agents such as RA. Such approaches have been shown to yield predominantly NC cells marked by HOX PG (1–7) members denoting the acquisition of a posterior cranial/vagal axial identity [74,82–85]. Using RA to posteriorise HOX negative cells requires optimisation as both the timing of RA addition and RA concentration appear to impact NC specification *in vitro* [86]. As such, although increasing concentrations of RA results in increased expression of posterior HOX genes, this occurs at the expense of NC markers such as SOX10 [83,86]. Similar to their *in vivo* counterparts, vagal NC cells generated through these protocols have been shown to further differentiate, via culture in neurotrophic media, toward various components of the ENS including distinct enteric neuronal subtypes marked by expression of 5HT, GABA and NOS [82,83,85,86]. Interestingly, cranial NC when treated in the same conditions, differentiated into TH positive neurons which are positive for TRKB, indicating that they preferentially give rise to sympathetic neurons [82]. Similarly, when cranial and vagal NC cells were grafted into the muscle wall of the mouse cecum at 3-6 weeks, vagal NC migrated and repopulated the host colon to a greater extent than cranial NC [82]. This finding highlights the importance of generating cell progenitors of the correct regional identity for potential use in clinical applications from hPSCs and demonstrates that early axial specification impacts cell functionality *in vivo*. Crucially, transplantation experiments revealed that hPSC-derived vagal NC/ENS progenitors can colonise and differentiate efficiently within the gut of host mice and even rescue the lethal aganglionic phenotype exhibited by *Ednrb* mutant mice, a model of Hirschsprung disease thus serving as a promising population for the development of cell therapy approaches against conditions affecting ENS specification [82,86].

Recent advances in organoid-based differentiation approaches have opened new avenues toward the establishment of more sophisticated *in vitro* models for dissecting NC function and related diseased states. To effectively study the complex role of ENS within the gastrointestinal (GI) tract, several studies have combined hPSC-derived vagal NC cells with human intestinal organoids (HIO), thereby generating an hPSC-derived assembloid model of intestinal tissue with functional ENS [82,84,87]. When combined, vagal NC progenitors generated glia and neurons of the ENS which integrated into the smooth muscle layer of the HIO and were found to promote cell proliferation in intestinal crypts and gene expression indicative of intestinal cell development. More recent work has described the generation of assembloid cultures containing colonic intestinal tissue with ENS and blood vessels; a model perhaps more suited to diseases which impact the distal part of the GI tract, including Crohn's and Hirschsprung's disease [88].

### Trunk NC

Although RA addition to cranial NC cells results in posteriorisation to a vagal axial identity, it does not lead to the induction of HOX genes posterior to HOX PG 7 [82,83,86] and early attempts to produce trunk NC derivatives from hPSCs have relied on the expression of PHOX2B or ASCL1, which also mark vagal NC derivatives in addition to trunk NC-derived sympathoadrenal progenitors [83,89]. A number of recent studies have demonstrated the efficient generation of NMP-like cells from both mouse and human PSCs following stimulation with Wnt (using the GSK-3 inhibitor CHIR99021 (CHIR) or Wnt3a) and FGF2/8 for 2–4 days [67,85,90–95]. Since then, extensive studies have optimised their generation and utilised these progenitors *in vitro* (reviewed in [20]). PSC-derived NMP-like cells have been shown to give rise to both neural spinal cord and paraxial mesoderm cells [1,93,96–100]. Interestingly, some studies have demonstrated that trunk NC can be generated via an NMP intermediate reflecting the developmental origin of these cells *in vivo* [85,91,101–105]. The earliest example of trunkNC induction and differentiation *in vitro* found that treating NMP-like cells with BMP resulted in up-regulation of NC markers and generated NC-like cells capable of generating peripheral nervous system (e.g. dorsal root ganglia and sympathetic ganglia) and Schwann cells and when transplanted to the mid/hindgut in quail migrated into the gut wall [102]. Subsequent studies, using a similar NMP cell induction method found that NC cells expressed trunk *HOX* genes and could be differentiated to sympathoadrenal progenitors and chromaffin cells [85,103,104,106]. Expanding these findings, Frith and colleagues demonstrated that a fraction of TBXT+ hPSC-derived NMPs expressed neural plate border genes and early neural crest markers (e.g. PAX3/7, MSX1/2 and ZIC1/3) [85]. Intermediate BMP and Wnt signalling then facilitated the production of high yields of SOX10+ NC-like cells from TBXT+ NMPs, which express HOX PG (6–9), the posterior spinal cord/NC marker CDX2 and can be differentiated to sympathoadrenal progenitors/sympathetic neurons [85,103]. Transplantations into the chick dorsal neural tube also showed the potential of these cells to integrate and migrate to the dorsal root ganglia recapitulating similar behaviour to their *in vivo* counterparts [85]. Interestingly, NMP-derived trunk NC cells have been reported to remain capable of generating adipocytes, chondrocytes and osteocytes [101,102]. This indicates either the co-emergence of minor cranial NC cell subpopulations or suggests a greater developmental potential of hPSC-derived trunk NC in line with data showing that *in vitro* cultured embryonic avian trunk NC exhibits the ability to generate some ectomesenchymal derivatives [107,108].

To achieve a homogeneous TBXT/SOX2-expressing NMP population and subsequent trunk NC formation, the intensity of Wnt stimulation requires titration to an intermediate to high level (CHIR; 3–12 µM) level [85,91,101,105]. The level required for NMP specification is higher than the low-intermediate level (<3 µM) activation which is required to generate cranial NC-like cells [101,105]. Increasing levels of Wnt stimulation also induce a higher expression of posterior *HOX* genes (e.g. HOXC9), most likely as a result of CDX up-regulation which drives posterior *HOX* gene expression and mediates NC induction [101,109–111]. Similarly to the fine tuning required to achieve correct BMP signalling, the specific level of CHIR required is dependent on each cell line, confluency of starting cell population and CHIR batch to batch variation, therefore, the unique balance of Wnt signalling requires optimisation in each differentiation protocol. Furthermore, the length as well as magnitude of Wnt signalling activation is also important in trunk NC specification as prolonged activation of Wnt signalling may drive mesoderm specification from NMPs [90], while combined Wnt/FGF signalling results in neural commitment [91,98,100].

FGF signalling is known to play a role in NMP specification and shaping axial identity, although some *in vitro* protocols omit FGF ligands indicating endogenous FGF signalling may be sufficient to induce an NMP identity [99,102,105,106]. Protocols which rely on CHIR alone or CHIR/FGF induced NMPs produce NC at a similar efficiency (Table 1). In axial progenitors, FGF signalling is required to pace the ‘HOX clock’ and mediates temporal HOX gene activation [92]. Similarly, inhibition of FGF during NC induction impairs HOX and CDX2 expression, although it does not impact SOX10 induction [101,105]. Conversely, high levels of FGF signalling may drive more sacral HOX expression (HOX PG (10–13)) in NC cultures at the expense of SOX10 expression [105]. Together, these data suggest that a certain level of FGF signalling activity is required to induce a trunk HOX identity and posterior markers such as CDX2 [101,105]. Transcriptomic analysis of NMPs and NMP-derived NC cells indicate that collinear *HOX* gene expression progresses over time in culture [85,91]. However, further work is required to define the signalling determinants of posterior axial identity/collinear *HOX* gene activation during NC specification from NMPs and more specifically whether this occurs in NMP/pre-neural progenitors, committed NC cells, or both.

A promising avenue in 3-dimensional (3D) cell culture models containing trunk NC derivatives, is the recent development of trunk neuromuscular organoids (NMOs) [112,113]. Using hPSC-derived NMPs aggregated to form 3D spheres, these bipotent progenitors are capable of generating mesodermal and ectodermal derivatives which self-organise into 3D organoids containing neuromuscular junctions (NMJ). As NMPs also contribute to trunk NC lineages, NMOs were found to contain Schwann cells which are essential for maturation and support of the neuromuscular junction *in vivo* and have been implicated in diseases such as amyotrophic lateral sclerosis [112,114]. Thus, this approach enables the analysis of the complex interaction of cells at the NMJ and the role of distinct cell types in disease onset and progression e.g. through the use of patient-derived induced pluripotent stem cell lines. More complex 3D models (known as gastruloids) reflecting early mammalian development have also been developed and these involve the induction of multiple interacting cell lineages [115–118]. These models have been shown to undergo transitions which recapitulate axis elongation and symmetry breaking events, somitogenesis, rudimentary neural tube formation and cardiogenesis [115–117,119,120]. Moreover, they have recently been shown to contain NC progenitors which differentiate to the peripheral nervous system and integrate with a primitive gut tube structure making them a useful model to study early human NC development [121].

### Sacral NC

To date sacral neural crest has received little attention in *in vitro* studies and has limited characterisation *in vivo*. Recent work *in vivo*, revealed that the trunk to tail transition in mouse embryos, is driven by a gene regulatory network centred around *GDF11*, *LIN28A* and *HOX13* genes [122–124]. GDF11, a member of the TGFβ signalling pathway, regulates *HOX* gene expression at the sacral-most level of the axis. In a negative feedback loop, acting to terminate axis elongation, up-regulation of *HOX13* genes inhibits *LIN28A* expression which results in cell cycle exit. In line with this, *in vitro* studies have demonstrated that GDF11 drives sacral HOX expression in derivatives of NMP-like cells *in vitro* [91–93]. Similarly, a rise in endogenous GDF11 signalling in mixed pre-neural progenitors/neural crest cultures was shown to induce sacral HOX expression thus facilitating the generation of neural crest with a sacral identity [91]. Further work to characterise these cells and their differentiation potential may yield an exciting sacral NC model with potential for therapeutic application and disease modelling for both the ENS and lower urogenital tract.

## Conclusions

**Figure 2.**
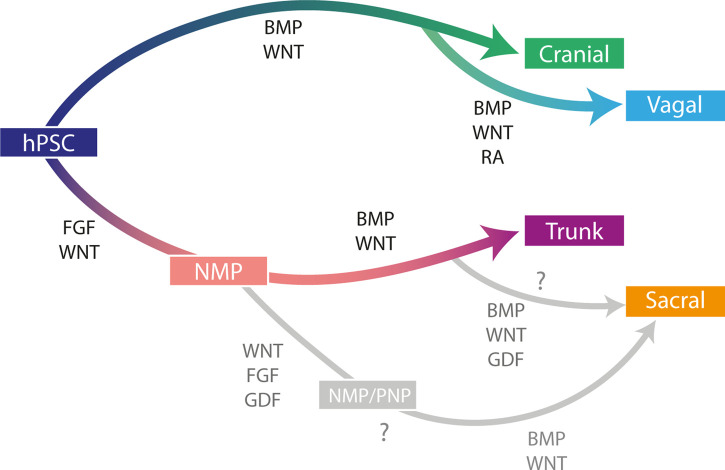
Anterior–posterior patterning in *in vitro* derived neural crest. Schematic diagram summarising the generation of the four subcategories of NC from human pluripotent stem cells (hPSCs). The major signalling pathways known to regulate differentiation are displayed for each subcategory. Cranial and vagal NC differentiation methods use the same initial signalling pathways but vagal NC are posteriorised using the addition of retinoic acid (RA) signalling. Trunk and sacral NC are derived via a common neuromesodermal progenitor (NMP) population. When sacral NC obtain a sacral identity remains unclear, but two potential routes and signalling pathways are displayed in grey. In the first route, trunk NC are posteriorized by GDF signalling, whereas in the second NMPs or pre-neural progenitors (PNP) are posteriorized by GDF prior to NC specification.

Over the last decade much progress has been made in developing human *in vitro* models of neural crest formation, aiding our understanding of the precise signalling pathways required to specify NC cells corresponding to all axial identities and consequently human neural crest biology (Table 1 and Figure 2). The success and advances in generating specific neural crest derivatives has also opened new opportunities to generate complex multilineage 3D models which mimic early development and exhibit complex multi-tissue interactions. Specifically, cranial NC cultures have demonstrated a potential application in tendon healing whereas vagal NC hold promise for treatment of ENS defects such as in Hirschsprung's disease [82,86,125]. However, further work is required to produce purified NC populations of defined regional identities, to understand the heterogeneity and potential of distinct NC populations and rigorously test their differentiation potential *in vitro* and *in vivo*. The increasing availability of single-cell transcriptomic datasets and 3D and 2D culture systems combined with improved fully defined differentiation protocols will significantly aid this goal.

**Table 1:** Published studies reporting the generation and axial characterisation, determined by HOX gene expression, of *in vitro* derived neural crest

Study	Axial identity based on *HOX* gene expression	Basal media	Differentiation protocol	SOX10 expression (%)	Differentiation potential (*in vitro*)
[74]	Cranial	KSR/N2 (DMEM:F12)	LDN (D0–3); SB43 (D0–4); 3 µM CHIR (D2–11)	53%	Melanocytes
[82]	Cranial	KSR/N2 (DMEM:F12)	SB43, LDN (D0–11); 3 µM CHIR (D2–11)	65%	Enteric neurons/glia
[101,126]	Cranial	B27 (DMEM:F12) + 0.5% BSA	2.5–3 µM CHIR, ROCKi (D0–2); basal media (D0–5)	60%	Osteocytes, chondrocytes, smooth muscle, adipocytes, peripheral neurons, glia and melanocytes
[76]	Cranial	B27 (DMEM:F12) + 0.5% BSA	3 µM CHIR (D0–5); ROCKi (D0–2)	63%	Chondrocytes, osteoblasts, peripheral neurons, glia and melanoblasts
[86]	Vagal	N2 (DMEM:F12)	SB43, DMH1, BMP4, 1 μM CHIR, (D0-6); ROCKi (D0-2); RA (D4-6)	40%	Enteric neurons/glia
[82]	Vagal	KSR/N2 (DMEM:F12)	SB43, LDN (D0-11); 3 μM CHIR (D2-11); RA (D6-11)	60%	Enteric neurons/glia
[74]	Vagal	KSR/N2 (DMEM:F12)	LDN (D0–3); SB43 (D0–4); 3 µM CHIR (D2–11); FGF or RA (various time points)	10–60%	Melanocytes
[83]	Vagal	KSR/N2 (DMEM:F12)	SB43 (D0–11); 3 µM CHIR (D0–11)	n.d. (20% SOX10E1^+^ or 20% PHOX2B^+^)	Sympathoadrenal cells and melanoblasts
[84]	Vagal	N2B27 (Neurobasal)/DMEM:F12 + insulin	FGF2, EGF (D0–6); RA (D4–6)	n.d. (99.5% p75NTR^+^/HNK-1^+^)	Enteric neurons/glia
[105]	Trunk	N2 (DMEM:F12)	3 µM CHIR, ROCKi (D0–2); BMP4 and DMH1 (D2–5)	80–90%	Melanocytes, peripheral neurons, sympathoadrenal, glia, osteocytes and smooth muscle
[101]	Trunk	B27 (DMEM:F12) + 0.5% BSA	7–12 µM CHIR, ROCKi (D0–2); basal media (D0–5)	75%	Osteoblasts, smooth muscle, peripheral neurons, glia, melanocytes and sympathoadrenal cells
[85]	Trunk	N2B27 (DMEM:F12, D0–3); N2 (DMEM:F12, D3–9)	FGF2 and 3–4 µM CHIR (D0–D3); SB42, DMH1, BMP4, 1 µM CHIR (D3–9)	60%	Sympathoadrenal progenitors/sympathetic neurons
[104]	Trunk	N2B27 (D1–5); ^1^NB (D5–D11)	3 µM CHIR, SB43 (D0–5); BMP2, FGF2 (D5–D11)	n.d. (98% p75NTR^+^/HNK-1^+)^	Sympathoadrenal cells/adrenomedullary chromaffin cells
[106]	Trunk	E6	2 µM CHIR, SB (D0–3); FGF2, BMP, RA (D3–10)	40–50%	Sympathetic neurons/glia
[91]	Trunk and Sacral	N2B27 -vitamin A (DMEM:F12)	FGF2, 3–5 µM CHIR, AGN, ROCKi (D0-36h); FGF2, 3–5 µM CHIR, AGN, ROCKi (36h — P10)	40%	Smooth muscle

The concentration of CHIR used in each experiment has been noted due to the role of Wnt signalling in mediated the cranial-trunk switch [101,105,127]. Only studies which have determined axial identity through *HOX* gene analysis have been included in this table [74,76,82–86,91,101,104–106,126].

1 NB, neurobasal [102]; CHIR, CHIR99021; D, day; P, passage; SB43, SB431542; ROCKi, ROCK inhibition; LDN, LDN193189; AGN, AGN193109; RA, retinoic acid.

## Perspectives

- NC can contribute to up to 47 cell types in humans [128]. NC differentiation potential is shaped by its A–P position, therefore defining and modulating A–P axial identity *in vitro* is a crucial aspect in the design of hPSC neural differentiation protocols.
- Cranial NC are specified from hPSCs through a pre-neural/neural plate border intermediate and under the influence of intermediate BMP activity and low Wnt signalling. RA signalling posteriorises cranial neural crest to a vagal identity but does not efficiently induce trunk NC. Instead, trunk NC specification relies on the generation of an NMP intermediate and subsequent intermediate BMP signalling. Generating sacral neural crest requires further work although GDF11 signalling may be critical in inducing a lumbosacral axial identity in hPSC neural derivatives.
- The therapeutic potential of each of these subgroups remains an exciting avenue to explore and calls for in depth understanding of heterogeneity of progenitor pool and their *in vivo* functionality.

## Open Access Statement

Open access for this article was enabled by the participation of University of Sheffield in an all-inclusive Read & Publish pilot with Portland Press and the Biochemical Society under a transformative agreement with JISC.

## Abbreviations

A–P: Anterior–posterior
CHIR: CHIR99021
ENS: enteric nervous system
hPSC: human pluripotent stem cells
NC: neural crest
NMPs: Neuromesodermal progenitors
PG: paralog groups
